# Chemokine receptor expression in tumour islets and stroma in non-small cell lung cancer

**DOI:** 10.1186/1471-2407-10-172

**Published:** 2010-04-29

**Authors:** Chandra M Ohri, Aarti Shikotra, Ruth H Green, David A Waller, Peter Bradding

**Affiliations:** 1 Department of Infection, Immunity and Inflammation, University of Leicester, Leicester, UK; 2 Institute for Lung Health, Department of Respiratory Medicine, Glenfield Hospital, Leicester, UK; 3 Department of Thoracic Surgery, Glenfield Hospital, Leicester, UK

## Abstract

**Background:**

We have previously demonstrated that tumour islet infiltration by macrophages is associated with extended survival (ES) in NSCLC. We therefore hypothesised that patients with improved survival would have high tumour islet expression of chemokine receptors known to be associated with favourable prognosis in cancer. This study investigated chemokine receptor expression in the tumour islets and stroma in NSCLC.

**Methods:**

We used immunohistochemistry to identify cells expressing CXCR1, CXCR2, CXCR3, CXCR4, CXCR5 and CCR1 in the tumour islets and stroma in 20 patients with surgically resected NSCLC. Correlations were made with macrophage and mast cell expression.

**Results:**

There was increased expression of CXCR2, CXCR3, and CCR1 in the tumour islets of ES compared with poor survival (PS) patients (p = 0.007, 0.01, and 0.002, respectively). There was an association between 5 year survival and tumour islet CXCR2, CXCR3 and CCR1 density (p = 0.02, 0.003 and <0.001, respectively) as well as stromal CXCR3 density (p = 0.003). There was a positive correlation between macrophage density and CXCR3 expression (r_s _= 0.520, p = 0.02) and between mast cell density and CXCR3 expression (r_s _= 0.499, p = 0.03) in the tumour islets.

**Conclusion:**

Above median expression of CXCR2, CXCR3 and CCR1 in the tumour islets is associated with increased survival in NSCLC, and expression of CXCR3 correlates with increased macrophage and mast cell infiltration in the tumour islets.

## Background

We have demonstrated previously that the tissue microlocalisation of immune cells in surgically resected NSCLC is a key determinant of patient survival [1,2]. In particular, when macrophages are located within tumour epithelial islets, a striking improvement in patient survival is evident. This supports the view that these islet-associated macrophages contribute to cytotoxic mechanisms which limit tumour dissemination. It is not known why some patients have high levels of potentially cytotoxic cells in their tumour islets and others do not, but the expression of macrophage-recruiting chemokines within the tumour islets by either tumour cells themselves or infiltrating immune cells is likely to be a key mechanism. Therefore an improved understanding of chemoattractant pathways that are active may lead to the development of novel chemotherapeutic agents.

Chemokines are cytokines that have chemotactic properties influencing cell migration [3]. Chemokines themselves are released in response to cell activation by diverse cytokines and pathologic stimuli [3]. The chemokine superfamily consists of over 40 ligands and approximately 20 receptors [3]. They can be classified into four groups: -CXC-, -CC-, -C- and -CX3C - according to amino acid position at the N terminal. Several chemokines have been shown to be present in cancer tissue including CCL2, CCL3, CCL4, CCL5, CCL8, CCL17, CCL18, CCL20, CCL21, CCL22, CCL27, CCL28, CXCL1, CXCL2, CXCL8, CXCL9, CXCL10, CXCL12, and CXCL13 [4-6]. An important ligand/receptor pair is CXCL12 and CXCR4 which may be involved in the regulation of metastasis in NSCLC [7]. CCL5 activates CCR1, -3 and -5, and CCL5 expression by tumour cells in patients with stage I lung adenocarcinoma has been associated with improved survival [8]. CXCL8 (interleukin-8) has two chemokine receptors, CXCR1 and CXCR2 [9]. CXCL8 is a mediator of angiogenesis in lung cancer [10] and correlates with angiogenesis, tumour progression and poor survival in NSCLC [11,12]. Zhu found that cell proliferation was significantly reduced by anti-CXCR1 antibody but not by anti-CXCR2 antibody and concluded that the mitogenic function of CXCL8 in lung cancer is mediated mainly by CXCR1 receptor [9]. However, it has also been demonstrated in a murine model that depletion of CXCR2 inhibits tumour growth and angiogenesis in lung cancer [13]. With respect to immune cell infiltration in cancer, CXCR3, along with IFN-γ, may play an important role with respect to NK cell infiltration [14].

The precise localisation of chemokine receptor expression in NSCLC with respect to tumour islets or stroma has not been examined. Given that we have demonstrated previously that the tissue microlocalisation of macrophages and mast cells in NSCLC is intimately related with outcome, [1,2], we hypothesised that this principle would also apply to the expression of chemokine receptors. If true, we would expect that patients with improved survival would have high tumour islet expression of chemokine receptors known to be associated with favourable prognosis in cancer such as CXCR3 and CCR1.

## Methods

### Study Population

The study was approved by the Leicestershire Research Ethics Committee. The tissue specimens evaluated were from 20 patients with NSCLC who had undergone resection with curative intent at the University Hospitals of Leicester National Health Service Trust (Leicester, United Kingdom). These patients had resections during two periods - one dating from 1991 to 1994 and the second from January to December 1999. This cohort of patients has been described previously [1]. Of the 20 patients studied, 14 were men and average age at surgery was 72.3 years (standard deviation, 6.53; range, 60.2 to 82.4 years). Full clinicopathologic information was gathered before and after surgery, including patient characteristics, treatment, combined clinical and surgical staging results (preoperative staging by computed tomography scan, selective mediastinoscopy, and systematic lymph node sampling at operation), histologic subtype, tumour grade, and survival data. Patients were divided into two groups, extended survival (ES) (mean ± SEM 90.8 ± 11.8 months) and poor survival (PS) (mean ± SEM 7.9 ± 0.8 months). Patient characteristics are shown in Table 1.

**Table 1:** Patient characteristics

Characteristic	Extended Survival	Poor Survival
No. of patients	10	10
		
Age - years	72.0 ± 2.5	72.6 ± 1.6
		
Male sex - no. (%)	8 (80)	6 (60)
		
Year of surgery - no. (%)		
1992	1 (10)	0 (0)
1994	0 (0)	1 (10)
1999	9 (90)	9 (90)
		
Tumour stage - no. (%)		
1	7 (70)	7 (70)
2	3 (30)	3 (30)
		
Histology - no. (%)		
Squamous	8 (80)	6 (60)
Adenocarcinoma	2 (20)	1 (10)
Large cell	0 (0)	1 (10)
Other	0 (0)	2 (20)
		
Tumour Grade - no. (%)		
Moderate	4 (40)	0 (0)
Poor	6 (60)	10 (100)
		
Adjuvant Chemotherapy (%)	0 (0)	0 (0)
		
Radiotherapy (%)	1 (10)	1 (10)
Palliative Radiotherapy (%)	1 (10)	1 (10)
		
Survival - months	90.8 ± 11.8	7.9 ± 0.8

Plus-minus values are means ± SEM

### Immunohistology

Specimens studied were formalin fixed and paraffin embedded. Only blocks containing the advancing edge of the primary tumour were evaluated. Tissue sections of 4 *μ*m thickness were cut onto glass slides and then de-waxed in xylene and rehydrated through graded alcohols. Antigen retrieval was carried out using Trilogy Antigen Retrieval solution (Cell Marque, Hot Springs, United States of America) in a pressure cooker (heated to 117.5°C for 1 min and then cooled to 100°C for 30 seconds). Mouse antihuman antibodies were used (all R & D Systems Europe, Abingdon, United Kingdom) as follows: CXCR1 (clone 42705), CXCR2 (clone 48311), CXCR3 (clone 49801), CXCR4 (clone 44716), CXCR5 (clone 51505) and CCR1 (clone 53504). Immunostaining was performed and the chemokine receptors were developed with peroxidase and 3,3'-diaminobenzidine tetrahydrochloride (brown reaction product). Sections were then counterstained with haematoxylin and mounted in an aqueous mounting medium (BDH Chemicals Ltd, Poole, United Kingdom). Appropriate isotype controls were performed where the primary antibodies were replaced by irrelevant mouse mAb of the same isotype and at the same concentration as the specific primary mAb.

### Analysis and Validation of Immunostaining

Analysis was performed blind with respect to the clinical outcome. The ten most representative high-power fields (x400) per slide were manually selected using an Olympus BX50 microscope (Olympus, Southall, United Kingdom). The respective areas of stroma and of tumour-cell islets were then measured at ×400 magnification using Analysis imaging software (Soft Imaging System GmbH). The number of nucleated macrophages and cells with positive staining for the phenotype marker in each area were then counted manually and expressed as cells/mm^2 ^of stroma or tumour islets. Analysis was repeated for 5 patients to assess repeatability and validity.

### Statistical Analysis

Statistical analyses were carried out using the GraphPad Prism software package (v. 4.02; GraphPad Prism Software Inc, San Diego, CA). For categoric analysis, the median value was used as a cut point to dichotomise the series. The χ^2 ^test was used to test for relationships between categoric variables, and the Mann-Whitney nonparametric test was used to compare categoric with continuous variables. Kaplan-Meier survival curves were used to look for correlations with survival and were compared with the use of the log-rank statistic. For the above comparisons, p < 0.05 was considered statistically significant.

## Results

### Patient Characteristics

Patent characteristics are shown in Table 1. Of the 20 patients studied, 15 had died at the time of analysis. Fourteen tumours were squamous, 3 adenocarcinoma, 2 large cell, and 1 other. Fourteen were stage I and 6 stage II. No patients had additional postoperative chemotherapy and 2 had additional radiotherapy for later palliation.

### Validation of Analysis

**Figure 1:**
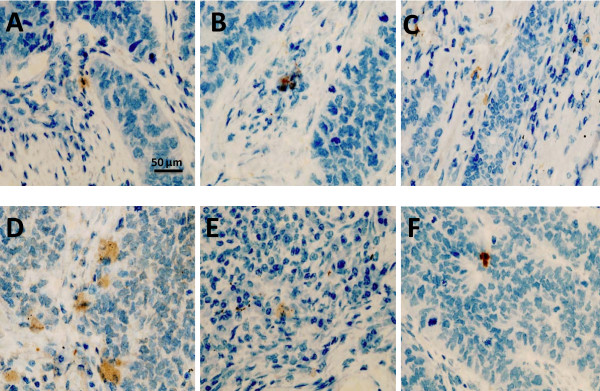
**Immunohistology demonstrating positive chemokines receptor expression (brown) for CXCR1 (A), CXCR2 (B), CXCR3 (C), CXCR4 (D), CXCR5 (E) and CCR1 (F)**. Magnification ×400.

Clear and distinguishable staining was evident for the chemokine receptors studied (Fig 1). Appropriate isotype controls were negative. In order to assess the validity of the method, area measurements and cell counts were repeated and intraclass correlation coefficients calculated. Good correlations were found for both: 0.997 (95% CI, 0.996 to 0.998, p < 0.001) and 0.995 (95% CI, 0.993 to 0.997, p < 0.001). This method of analysis has also been validated by our group previously [1,2].

### Cellular Distribution

**Figure 2:**
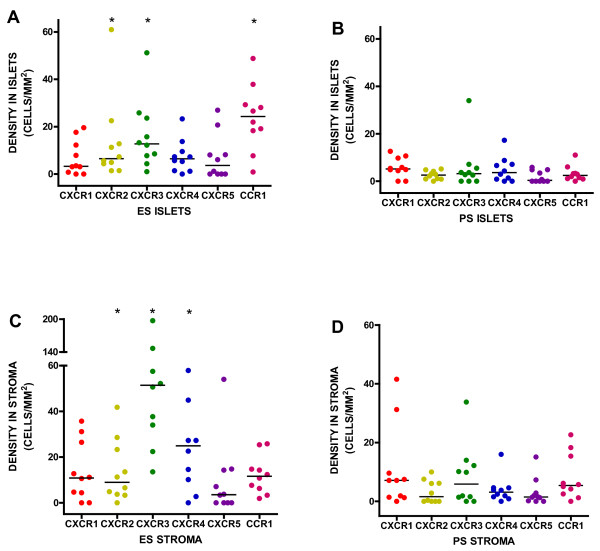
**Chemokine receptor densities in the tumour islets for extended survival (ES) patients (A) and poor survival (PS) patients (B) and in the stroma for ES patients (C) and PS patients (D)**.

The majority of chemokine receptor immunostaining in the tumour islets was present in inflammatory cells and rarely seen on tumour epithelial cells. Similarly in the stroma, immunostaining was also seen in inflammatory cells with no expression evident on vessels. There was significantly increased expression of CXCR2, CXCR3, and CCR1 in the tumour islets of ES compared with PS patients [median 6.5 versus 2.6, (p = 0.007), 12.7 versus 3.2, (p = 0.01), and 24.3 versus 2.4 cells/mm^2^, (p = 0.002), respectively]. There were no significant differences for CXCR1, CXCR4, or CXCR5 in either the tumour islets or stroma (Figs 2A and 2B). There was significantly increased expression of CXCR2, CXCR3 and CXCR4 in the stroma of ES compared with PS patients (8.9 versus 1.6, (p = 0.04), 51.4 versus 5.9, (p < 0.001), 24.9 versus 3.1, (p = 0.004) (Figs 2C and 2D).

### Kaplan-Meier Survival Analysis

**Figure 3:**
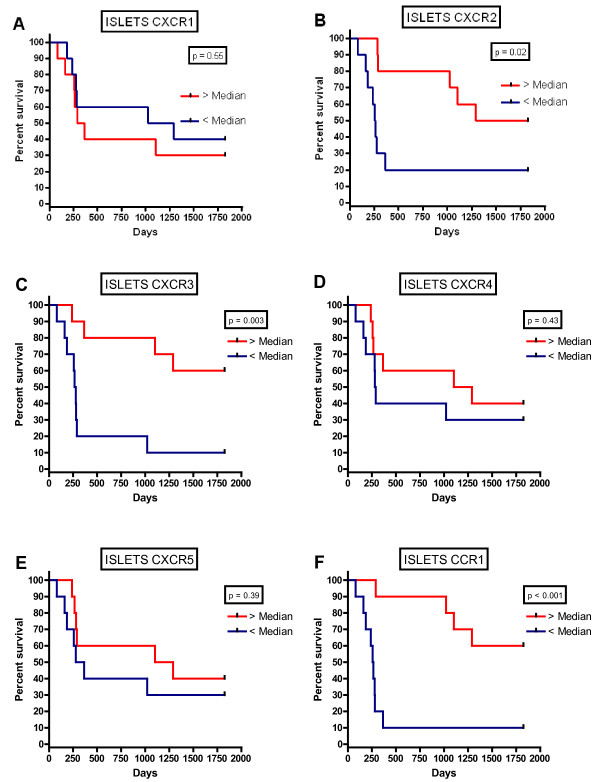
**Kaplan-Meier five year survival curve for chemokines receptor densities in the tumour islets for CXCR1(A), CXCR2 (B), CXCR3 (C), CXCR4 (D), CXCR5 (E) and CCR1 (F)**.

**Figure 4:**
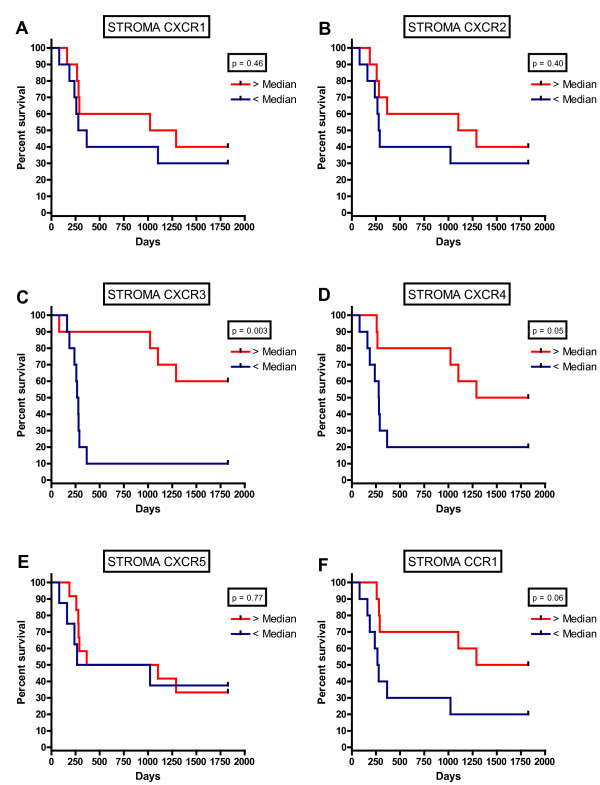
**Kaplan-Meier five year survival curve for chemokines receptor densities in the tumour stroma for CXCR1(A), CXCR2 (B), CXCR3 (C), CXCR4 (D), CXCR5 (E) and CCR1 (F)**.

For further analysis, the data were divided into two groups above and below the median cell count values. Kaplan-Meier survival curves were plotted to investigate further the association of cell densities with survival. The logrank statistic was used to compare survival rates. There was a positive association between survival and tumour islet CXCR2 (p = 0.02), CXCR3 (p = 0.003) and CCR1 (p < 0.001) density, but no significant associations between survival and tumor islet CXCR1, CXCR4 and CXCR5 density (Fig 3A-F). After dichotomisation at the median cell density for cells expressing each marker, 5-year survival was 50.3% above the median compared with 19.8% below the median for CXCR2, 60.2% versus 9.9% for CXCR3 and 59.8% versus 9.9% for CCR1. There was a positive association between survival and stromal CXCR3 (p = 0.003) density, but no significant associations between survival and stromal CXCR1, CXCR2, CXCR4, CXCR5 and CCR1 density (Fig 4A-F).

### Correlation with macrophage and mast cell counts

**Figure 5:**
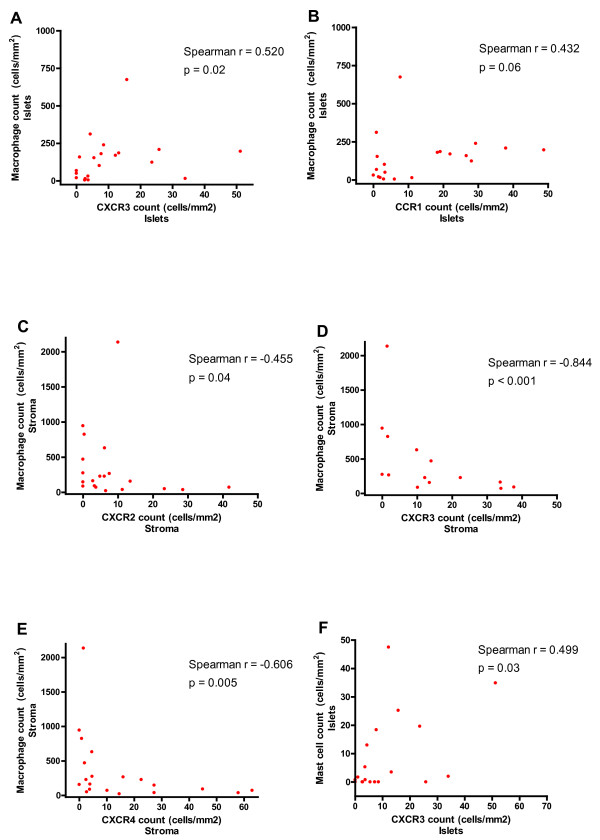
**Correlations between chemokine receptor and macrophage cell counts in the tumour islets for CXCR3 (A) and CCR1 (B), in the tumour stroma for CXCR2 (C), CXCR3 (D), and CXCR4 (E), and between mast cells and CXCR3 (F) in the tumour islets**.

Chemokine receptor expressing cell counts were correlated against islet and stromal macrophage counts which had been generated previously [1]. It was noted that there were positive correlations for CXCR3 (r_s _= 0.520, p = 0.02) and CCR1 (r_s _= 0.432, p = 0.06) in the islets (Fig 5A and 5B). With respect to stromal counts there were negative correlations for CXCR2 (r_s _= -0.455, p = 0.04), CXCR3 (r_s _= -0.844, p < 0.001) and CXCR4 (r_s _= -0.606, p = 0.005)(Fig 5C-E).

Chemokine receptor expressing cell counts were correlated against islet and stromal mast cell counts which had been generated previously [1]. There was a positive correlation for CXCR3 (r_s _= 0.499, p = 0.03) in the islets (Fig 5F).

## Discussion & Conclusions

The purpose of this study was to investigate the expression of chemokine receptors and their microlocalisation in surgically resected NSCLC. The results demonstrate that patients with extended survival have significantly increased expression of CXCR2, CXCR3 and CCR1 in their tumour islets compared to patients with poor survival, and significantly increased expression of CXCR2, CXCR3 and CXCR4 in their tumour stroma. In addition, we observed that patients with above median expression of CXCR2, CXCR3 and CCR1 in their tumour islets and patients with above median expression of CXCR3 in their stroma had significantly improved 5-year survival. Interestingly, when chemokine receptor expression was compared with macrophage density in the tumour islets (Fig 5), it was noted that there was a positive correlation with increasing islet macrophage count and expression of CXCR3 and CCR1 and also, between islet mast cell numbers and CXCR3.

We have demonstrated previously the importance of cellular microlocalisation in terms of potential sites for immune cytotoxicity against NSCLC tumours [1,2]. In particular, NSCLC tumour islets are the likely site of host cytotoxic responses against tumour development and progression. Not only does increasing macrophage infiltration of NSCLC tumour islets correlate strongly with survival, but these macrophages are predominantly of the M1 cytotoxic phenotype [2]. Our current results suggest that the chemokine receptor CCR1 which is expressed by macrophages, may be involved in the chemotactic pathway which recruits M1 macrophages into the tumour islets. Of particular relevance to this, a CCR1 ligand CCL5, is over-expressed in NSCLC tumours characterized by T lymphocyte infiltration [8]. Of further relevance, one of the ligands for CCR1 is CCL3 which is thought to stimulate the production and release of TNFα, a cytokine that is expressed and which has cytotoxic potential in the tumour islets [2].

CXCR3 has not been described on macrophages, but is expressed on cytotoxic NK cells [14], human lung mast cells [15] and T lymphocytes [16]. These cell types are associated with improved survival in NSCLC [1,17,18], and are likely to interact with macrophages in mediating tumour killing. CXCR3 expression was markedly increased in both the tumour islets and stroma in NSCLC patients with extended survival compared to those with poor survival. Tumours enriched for cells expressing CXCR3 are likely to be producing excess quantities of one or all of the CXCR3 ligands, CXCL9, -10 and -11. CXCL10 has been shown to be expressed by tumour epithelial cells in squamous NSCLC [19]. CXCR3 agonists protect against tumour dissemination in mouse models [19-21], observations supported by the improved outcome associated with increased CXCR3 expression in this study. In mouse models, CXCR3 ligands are thought to work predominantly through inhibition of angiogenesis, but our findings of CXCR3 expression on numerous inflammatory cells, suggests an important role in the host anti-tumour immune response. The CXCR3 ligands can be produced by a number of inflammatory and structural cells, and are typically thought of as markers of a Th1 immunological response as they are induced predominantly by IFNγ and TNFα [22]. Overall, the increased expression of CXCR3 on inflammatory cells within the NSCLC tumour stroma and islets in patients with extended survival, supports the view that cytotoxic cell-mediated immunity contributes to protection of the host against NSCLC progression, and suggests that augmenting this arm of the immunological response may be beneficial clinically.

Examining the expression and cellular provenance of the ligands for CXCR3 and CCR1 will be important in future studies of NSCLC. CCR1 ligands can also be produced by a variety of immune and structural cells, and CCL5 is produced by NSCLC tumour epithelial cells [8]. It is attractive to speculate therefore that the key factor determining the nature, intensity and microlocalisation of the immune response in NSCLC is the pattern and intensity of chemokine production by the tumour itself. Our finding of distinct chemokine receptor patterns in the tumour and stroma which associate with survival supports this view.

The chemokine receptor CXCR4 is implicated in the regulation of cancer growth, metastasis, relapse and response to treatment. In patients with NSCLC, CXCR4 expression has been found on circulating cytokeratin+ cells, with increased expression correlating with improved survival [23]. Our results suggest that increased expression of CXCR4 in the NSCLC stroma relates to improved survival. From our previous investigations [1,2], however, the tumour islets appears to be the critical micro-compartment for host immune responses against NSCLC. Further work will therefore be required to understand the biological role of stromal CXCR4 expression.

The chemokine receptor CXCR2 exerts multiple functions depending on the cell type it is expressed on. In NSCLC it is generally considered to promote tumorigenesis and metastasis through the induction of angiogenesis [9,13]. However, it is also implicated in the senescence of pre-neoplastic cells and may inhibit neoplastic transformation [24]. In our NSCLC tissue samples, CXCR2 in the tumour islets was not seen on tumour epithelial cells but was present on inflammatory cells. Interestingly, its expression was increased in the tumour islets of patients with extended survival compared to those with poor survival. This is compatible with the suggestion that loss of CXCR2 on tumour epithelial cells promotes neoplastic transformation in NSCLC [24], but also suggests that CXCR2 may serve to limit tumour growth through its expression on inflammatory cells recruited to the tumour. Thus CXCR2 may have dual roles in NSCLC, promoting tumour progression through the induction of angiogenesis in the stroma, but limiting tumour growth through the recruitment of inflammatory cells to the tumour islets. Targeting CXCR2 in NSCLC may therefore prove to have unpredictable effects depending on the relative balance between these two opposing activities.

In summary, above median expression of the chemokine receptors CXCR2, CXCR3 and CCR1 in the tumour islets is associated with increased 5-year survival. Increased expression of CXCR3 and CCR1 correlates with increased macrophage expression in the tumour islets and therefore these receptors may be involved in the pathway that attracts cytotoxic M1 macrophages, mast cell, B cells, T cells and NK cells into the tumour islets. Therefore, if a therapeutic agent could increase the production of the ligands for CXCR3 and CCR1 by the tumour epithelial cell, this might lead to an improved immune response to NSCLC and thus better survival.

## Competing interests

The authors declare that they have no competing interests.

## Authors' contributions

CO carried out the immunohistochemical staining, slide analysis, statistical analysis and prepared the manuscript. AS assisted with microtomy and the immunohistochemical staining. RG carried out the statistical analysis. DW provided the samples for analysis and prepared the manuscript. PB conceived the study, participated in its design and coordination, and prepared the manuscript. All authors have read and approved the final manuscript.

## Pre-publication history

The pre-publication history for this paper can be accessed here:

http://www.biomedcentral.com/1471-2407/10/172/prepub
